# *In vivo* bioluminescence imaging for viable human neural stem cells incorporated within *in situ* gelatin hydrogels

**DOI:** 10.1186/s13550-014-0061-3

**Published:** 2014-11-12

**Authors:** Do Won Hwang, Kyung Min Park, Hye-kyung Shim, Yeona Jin, Hyun Jeong Oh, So Won Oh, Song Lee, Hyewon Youn, Yoon Ki Joung, Hong J Lee, Seung U Kim, Ki Dong Park, Dong Soo Lee

**Affiliations:** Department of Nuclear Medicine, Seoul National University College of Medicine, 28 Yongon-Dong, Jongno-Gu, Seoul, 110-744 Republic of Korea; Molecular Medicine and Biopharmaceutical Science, Graduate School of Convergence Science and Technology, and College of Medicine or College of Pharmacy, Seoul National University, Seoul, Republic of Korea; Department of Molecular Science and Technology, Ajou University, 5 Woncheon, Yeongtong, Suwon, 443-749 Republic of Korea; Cancer Research Institute, Seoul National University College of Medicine, Seoul, Republic of Korea; Cancer Imaging Center, Seoul National University Cancer Hospital, Seoul, Republic of Korea; Medical Research Institute, Chung-Ang University College of Medicine, Seoul, Republic of Korea; Division of Neurology, Department of Medicine, University of British Columbia, Vancouver, BC Canada

**Keywords:** Gelatin-based hydrogel, Matrix elasticity, Human neural stem cell, *In vivo* bioluminescence imaging, Optical kinetics

## Abstract

**Background:**

Three-dimensional (3D) hydrogel-based stem cell therapies contribute to enhanced therapeutic efficacy in treating diseases, and determining the optimal mechanical strength of the hydrogel *in vivo* is important for therapeutic success. We evaluated the proliferation of human neural stem cells incorporated within *in situ*-forming hydrogels and compared the effect of hydrogels with different elastic properties in cell/hydrogel-xenografted mice.

**Methods:**

The gelatin-polyethylene glycol-tyramine (GPT) hydrogel was fabricated through enzyme-mediated cross-linking reaction using horseradish peroxidase (HRP) and hydrogen peroxide (H_2_O_2_).

**Results:**

The F3-effluc encapsulated within a soft 1,800 pascal (Pa) hydrogel and stiff 5,800 Pa hydrogel proliferated vigorously in a 24-well plate until day 8. *In vitro* and *in vivo* kinetics of luciferase activity showed a slow time-to-peak after d-luciferin administration in the stiff hydrogel. When *in vivo* proliferation of F3-effluc was observed up to day 21 in both the hydrogel group and cell-only group, F3-effluc within the soft hydrogel proliferated more vigorously, compared to the cells within the stiff hydrogel. Ki-67-specific immunostaining revealed highly proliferative F3-effluc with compactly distributed cell population inside the 1,800 Pa or 5,800 Pa hydrogel.

**Conclusions:**

We examined the *in vivo* effectiveness of different elastic types of hydrogels encapsulating viable neural stem cells by successfully monitoring the proliferation of implanted stem cells incorporated within a 3D hydrogel scaffold.

**Electronic supplementary material:**

The online version of this article (doi:10.1186/s13550-014-0061-3) contains supplementary material, which is available to authorized users.

## Background

Rapid advances in stem cell-based therapy have opened up the possibility of stem cell use to reconstitute injured tissues for the functional improvement in the clinic. Particularly, neural stem cells, capable of being differentiated into functional neurons, could become a good cell source for the treatment of neurodegenerative diseases [1–4]. In spite of this progress, studies concerning stem cell therapy have shown poor survival rates for the implanted stem cells, owing to the necrotic environment of the injured and inflamed tissues. This remains a critical limitation for successful cell therapy. To overcome this challenge, a variety of biomaterials such as microfiber-type or gel-type scaffolds have been developed to support survival and proliferation of implanted stem cells [5–11].

Among the many scaffolds currently available, hydrogels, capable of imbibing large amounts of water and possessing suitable physicochemical properties, are known to exhibit the best biocompatibility and biodegradability *in vivo*. The hydrogel materials can be fabricated by various chemical and physical reactions, and their physiochemical properties, such as gelation, mechanical properties, and degradation time, could be easily controlled [12,13]. In addition, the hydrogels can encapsulate therapeutic drugs and cells for the treatment of targeted diseases as they are formed in mild physiological conditions. These characteristics facilitated the use of hydrogels in therapeutic cell implants or as therapeutic delivery vehicles [14–16]. Hydrogels, which are cross-linked hydrophilic polymers, can serve as a bio-artificial niche not only for promoting survival of the implanted cells, but also for protection of the cells from the damaged tissue environment *in vivo*, by providing mechanical framework and three-dimensional superporous structures. The matrix hydrogel elasticity could be adjusted to match the stiffness of real tissues (brain: 0.1 to 1 kilopascal [kPa]; muscle: 8 to 17 kPa; collagenous bone: >34 kPa) and optimized to induce specific cell fates from the implanted mesenchymal stem cells [17–19]. Furthermore, studies have shown that hydrogel-encapsulated cells were functionally effective in several disease models [11,20,21]. Despite this effectiveness of the biomimetic hydrogel, the characteristics of different *in vivo* hydrogels are not understood for their real *in vivo* behavior of hydrogel-encapsulated cells. An *in vivo* imaging technique that tracks the survival of implanted stem cells within the hydrogel will help evaluate the *in vivo* efficacy of different hydrogel matrix types.

The gelatin-polyethylene glycol-tyramine (GPT) hydrogel, recently developed in our group, is an *in situ* cross-linkable hydrogel that exhibits rapid gel formation induced by the cross-linking reaction of horseradish peroxidase (HRP) with hydrogen peroxide (H_2_O_2_) [22]. This enzyme-mediated type of hydrogel possesses significant advantages of excellent biocompatibility and controllable mechanical strength. In addition, because this hydrogel is compatible with an injection system that can easily be applied *in vivo*, with minimal surgical invasiveness, it has the potential for use in tissue engineering-based regenerative therapy [22,23].

Bioluminescence light is emitted from the catalytic reaction of luciferase enzyme with its substrate, and *in vivo* administration of d-luciferin can be used to generate bioluminescence in implanted luciferase-expressing stem cells encapsulated within the hydrogel in small animals. The permeability of d-luciferin within the hydrogel may differ according to its mechanical strength. Therefore, examining the *in vivo* kinetics of the luciferase activity in the living mouse bearing the hydrogel-encapsulated stem cells after d-luciferin administration is necessary to acquire the optimal bioluminescence signal in implanted stem cells within hydrogels of different elasticity.

In this study, we investigated the survival and proliferation of injectable hydrogel-encapsulated stem cells by non-invasively monitoring human neural stem cells carrying the highly sensitive luciferase gene. Based on this *in vivo* imaging strategy, cell survival and proliferation in soft and stiff hydrogels were evaluated in nude mice with analysis of *in vivo* kinetics of the luciferase substrate.

## Methods

### Synthesis of GPT conjugate

In our previous report, the GPT hydrogel was developed as an injectable material with excellent biocompatibility and bioactivity for tissue regeneration and drug delivery [22]. The GPT conjugate was synthesized by coupling tyramine (TA)-conjugated polyethylene glycol (PNC-PEG-TA) and gelatin. Briefly, the hydroxyl groups of polyethylene glycol (PEG) reacted with *p*-nitrophenyl chloroformate (PNC) to activate the terminal groups (PNC-PEG-PNC), and then the PNC-PEG-PNC reacted with TA and gelatin to give the GPT polymer. The chemical structure and degree of substitution (DS) of TA were characterized by ^1^H NMR and UV measurements (^1^H NMR (D_2_O): δ 4.8 (m, the proton of anomeric carbon of gelatin), δ 0.8 to 4.6 (m, alkyl proton of gelatin), δ 3.5 to 3.8 (m, -CH_2_-CH_2_ of PEG ethylene), and δ 6.8 and 7.1 (m, aromatic protons of TA)). The TA content in the polymer was determined by UV-vis spectroscopy measurements at a wavelength of 275 nm. Utilizing a tyramine standard curve, we found that the TA content in the GPT conjugate was 110 μmol/g of GPT polymer. Gelatin (type A from porcine skin, >300 Bloom), PEG (MW 4,000), HRP (250 to 330 units/mg solid), aqueous hydrogen peroxide (H_2_O_2_; 30% [*w*/*w*]), 4-dimethylamino pyridine (DMAP), and PNC were purchased from Sigma-Aldrich (St. Louis, MO, USA). TA was obtained from Acros Organics (Geel, Belgium). Triethylamine (TEA) was supplied by Kanto Chemical Corp. (Tokyo, Japan). Aluminum oxide (Al_2_O_3_) was purchased from Strem Chemicals (Newburyport, MA, USA). Other chemical reagents and solvents were used without further purification.

### Preparation of the hydrogels and measurement of the elastic modulus (*G′*)

The GPT hydrogels (200 μL) were prepared through enzyme-mediated reaction using HRP and H_2_O_2_. One-hundred microliters of GPT polymer solution (3% *w*/*w*) dissolved in HRP stock solution and another 100 μL dissolved in H_2_O_2_ solution (0.0038% to 0.0075% w/w) were mixed to form the hydrogels. All solutions were dissolved in 0.01 M phosphate-buffered saline (PBS; pH 7.4). The elastic modulus (*G′*) was measured with an Advanced Rheometer GEM-150-050 (Bohlin Instruments, East Brunswick, NJ, USA) using the parallel plate (20-mm diameter) configuration at 37°C in oscillatory mode. The GPT polymer was dissolved both in HRP solution (0.0005 mg/mL of stock solution) and in solutions containing different concentrations of H_2_O_2_ (0.0038% to 0.0075% w/w of stock solution). Two-hundred microliter polymer solutions containing HRP and H_2_O_2_ were rapidly mixed on the bottom plate of the instrument, and the upper plate was immediately lowered down to a measuring gap size of 1 mm. A frequency of 0.1 Hz (single frequency) and a strain of 0.1% (strain control) were applied for the analysis to maintain a linear viscoelastic response.

### Cell culture and effluc virus infection

Use of fetal brain tissue collected for research was approved by the Clinical Research Screening Committee and the Internal Review Board (IRB) of the University of British Columbia (for preparation of an immortalized human neural stem cell line used in the present study). V-myc oncogene-harbored F3 human neural stem cell lines derived from 15-week-old fetal telencephalon periventricular layers [24,25] were maintained in Dulbecco's modified Eagle's medium (DMEM, Invitrogen, Grand Island, NY, USA) supplemented with 10% (v/v) fetal bovine serum (Invitrogen, Grand Island, NY, USA) with 10 U/mL penicillin and 10 μg/mL streptomycin (Invitrogen, Grand Island, NY, USA) in a humidified incubator at 37°C.

For *in vivo* visualization of grafted stem cells, F3 cells were genetically engineered using a retroviral vector (kindly provided by Dr. Brian Rabinovich of MD Anderson Cancer Center). The backbone of the retroviral MSCV DNA vector contains the enhanced firefly luciferase coding gene (effluc; modified by the codon optimization technique) and Thy1.1 (CD90.1), which is linked with IRES (internal ribosome entry site) and regulated by the cytomegalovirus (CMV) promoter in the 5′-LTR (long terminal repeat) region. For retrovirus production, the viral polyproteins (gag, pol, and env) were transfected into 293FT packaging cells. The F3 cells were infected with the harvested viral supernatant in the presence of 10 mM polybrene to prevent electrostatic repulsion between the virus and cell membrane. F3 cells transfected with the enhanced firefly luciferase gene (F3-effluc) were separated by magnetic-activated cell sorting (MACS) (Miltenyi Biotec Ltd., Bisley, Surrey, UK) using monoclonal anti-CD90.1 microbeads. The purity of magnetically separated CD90.1+ F3-effluc cells was examined by fluorescence-activated cell sorting (FACS) analysis (BD Immunocytometry System, Becton Dickinson, Franklin Lakes, NJ, USA) using the monoclonal antibody anti-CD90.1 conjugated to fluorescein isothiocyanate (FITC).

### *In vitro* bioluminescence assay

After F3-effluc cells were mixed with hydrogels possessing matrix strengths of 1,800 Pa (GPT 1.8 K) or 5,800 Pa (GPT 5.8 K), the firefly luciferase activity was measured in a 96-well plate (*n* = 3). *In vivo*d-luciferin (0.1 mL at 3 μg/μL) was directly treated into the cell/hydrogel mixture. Simultaneous treatment of each group with d-luciferin was carried out using a multipipette. The luciferase intensity was measured using a microplate luminometer (TR717, Applied Biosystems, Carlsbad, CA, USA) with an integration time of 20 s. The kinetics of the luciferase activity after d-luciferin administration was examined *in vitro* using a microplate luminometer for a period of 95 min with 5-min intervals between measurements.

### *In vivo* bioluminescence imaging in cell/hydrogel-bearing nude mice

Mice were maintained without unnecessary pain or distress, and 8-week-old male BALB/c nude mice were used for the *in vivo* hydrogel study. All animals were housed under specific pathogen-free animal conditions and handled in accordance with the ethical and biosafety guidelines. This protocol was approved by the Institutional Animal Care and Use Committee (IACUC NO. 09-0087) of Seoul National University Hospital. In our pilot study, we injected two types of GPT hydrogels (1.8 and 5.8 kPa) to confirm their *in vivo* durability in the subcutaneous region. Injected GPT hydrogels (1.8 kPa) degraded completely within 1 week, whereas GPT hydrogels (5.8 kPa) remained intact (data not shown). We prepared 5 × 10^5^ F3-effluc cells as pellets in centrifugation at 1,500 rpm for 5 min, which were then mixed with GPT solution containing HRP. A dual hydrogel injection system with two separate syringes filled with HRP (25 μg/mL) dissolved in GPT solution A or in H_2_O_2_ (8 μg/mL) dissolved in GPT solution B was used for *in situ* formation of the cell-hydrogel complex. A 1-mL syringe was filled with the mixture of F3-effluc cells and GPT solution A (+HRP), and the other was filled with the F3-effluc/GPT solution B (+H_2_O_2_) mixture. After each GPT solution was filtered using a 200-nm pore-size syringe filter, they were added into the dual injection syringe, and using a 26-gauge needle, they were subcutaneously loaded into the thigh of the nude mice.

To acquire the bioluminescence images, the mice (*n* = 3) were anesthetized with 2% isoflurane at low rates of O_2_ gas flow (1 L/min) via a nose cone after initial exposure inside an induction chamber. For *in vivo* bioluminescence imaging, d-luciferin was administered via intraperitoneal injection at a dosage of 150 mg/kg. The bioluminescence images were acquired using an IVIS-100 equipped with a highly sensitive CCD camera (Caliper Life Sciences, Hopkinton, MA, USA). Bioluminescence light emission was measured and integrated over 5 min. Region of interest (ROI) signal intensity was measured in each representative area and expressed as photon · s^−1^ · cm^−2^ · steradian^−1^. IVIS-100 bioluminescence image parameter mode for binning and f/stop was set up using values of 2 and 1, respectively.

### Immunohistochemistry analysis to investigate the *in vivo* characteristics of implanted hydrogel-encapsulated F3-effluc cells

Each sample resected from the cell-only, GPT 1.8 K, and GPT 5.8 K groups was sectioned (8-μm-thick sections). Fixed tissues were prepared using 4% formaldehyde at room temperature for 20 min and were washed twice for 10 min using PBS. To perform each step of the blocking and permeabilization procedure simultaneously, the mixture of 20% normal goat serum (NGS) and 0.5% Triton X-100 in 0.01 M PBS was added to each slice for 20 min. The permeabilized samples were incubated with primary antibodies specific for Tuj1 (Cell Signaling, Danvers, MA, USA) and Ki-67 (Abcam, MA, USA) overnight at 4°C. After each sample was rinsed three times using 0.01 M PBS for 5 min, they were incubated with fluorescence-labeled (Alexa Flour) secondary antibodies for 60 min. The slices were mounted on cover slips with an aqueous mounting medium supplemented with 4′,6-diamidino-2-phenylindole dihydrochloride (DAPI) (Vector Laboratories Inc., Burlingame, CA, USA).

### Statistical analysis

Data are shown as means ± standard error of the mean (SEM) and were calculated using Student's *t* test. Statistical significance was accepted at *P* values of less than 0.1.

## Results

### *In situ* cross-linkable GPT hydrogels and their mechanical strength

**Figure 1:**
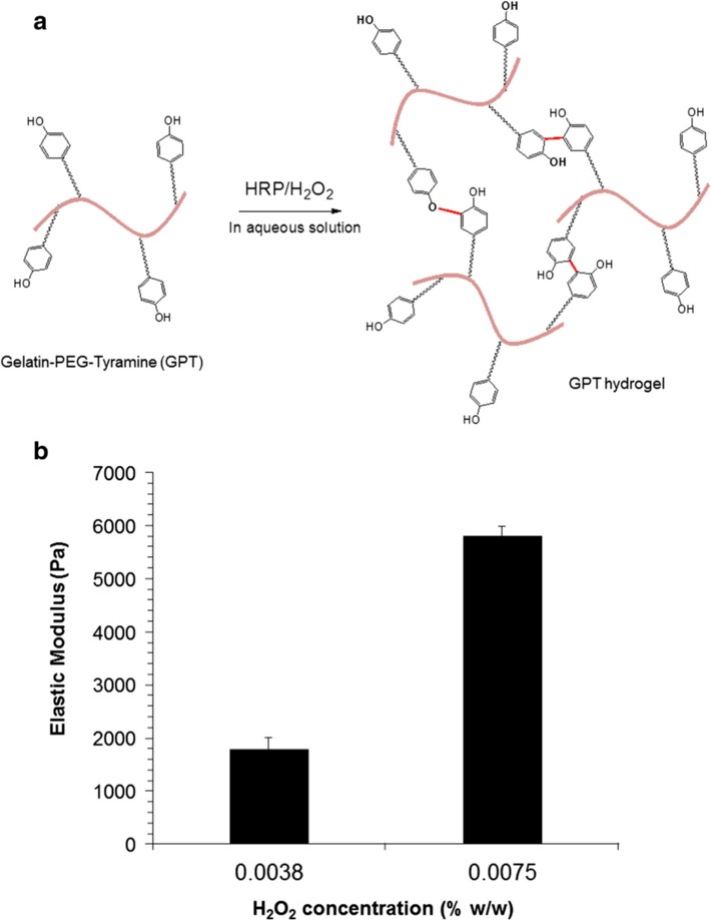
**Scheme of the** ***in situ*** **formation of gelatin-polyethylene glycol-tyramine (GPT) hydrogel. (a)** The *in situ* formation of GPT hydrogels occurred via an enzyme-mediated reaction using horseradish peroxidase (HRP) and H_2_O_2_ in an aqueous solution. **(b)** Elastic modulus (*G′*) of the GPT hydrogels (3% *w*/*w*) was measured with different H_2_O_2_ concentrations (0.0038% to 0.0075% *w*/*w*).

The GPT hydrogels were formed via an enzyme-triggered coupling reaction using HRP and H_2_O_2_. Figure 1a shows the enzyme-mediated hydrogel formation of GPT conjugates. The phenol molecules of the GPT graft copolymer are conjugated to each other via the carbon-carbon bond at the ortho positions or via a carbon-oxygen bond between the carbon at the ortho position and the phenoxy oxygen, by an enzyme-triggered oxidative reaction.

The measurement of elastic modulus (*G′*) of GPT hydrogels in a time-controlled oscillatory mode with different H_2_O_2_ concentrations revealed higher *G′* values (5,800 Pa) of the GPT hydrogels with higher H_2_O_2_ concentration (0.00075% *w*/*w*) because H_2_O_2_ plays as a cross-linker in the enzyme-triggered cross-linking system. When two different solutions (solution A contains HRP and solution B contains H_2_O_2_) were rapidly mixed together with a ratio of 1:1, the reaction mixture started to form GPT hydrogel. Hereafter, we called the hydrogel with 1,800 Pa (GPT 1.8 K) the soft hydrogel and that with 5,800 Pa (GPT 5.8 K) the stiff hydrogel.

### Morphological difference of F3-effluc cells in the soft and the stiff GPT hydrogels

**Figure 2:**
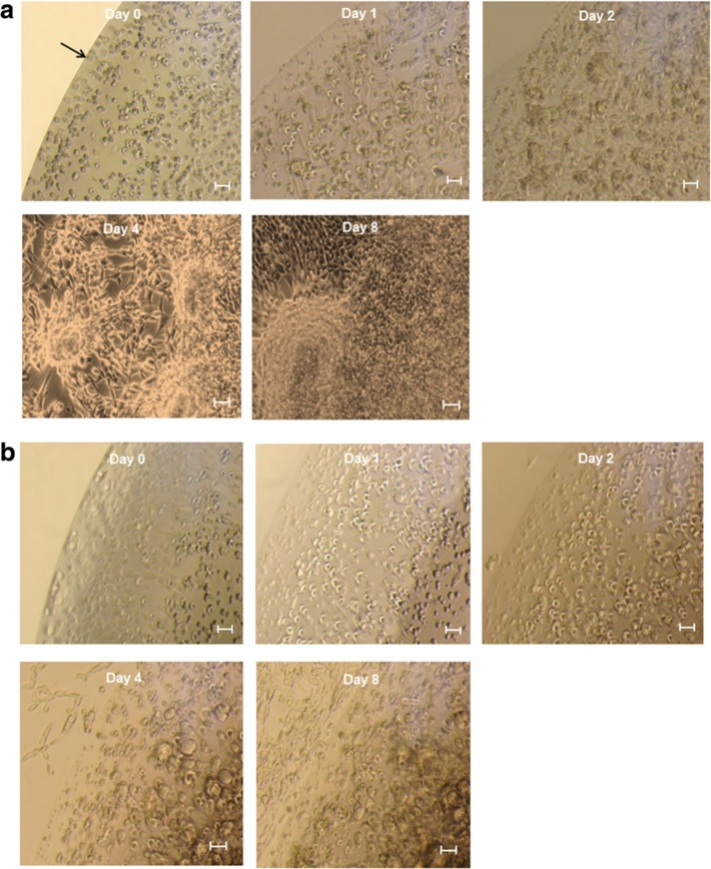
**Three-dimensional culture of human neural stem cells, F3-effluc, within the soft and stiff GPT hydrogels. (a)** In the bottom of the 24-well plate, 20 μL of cell/hydrogel was added (1:1 ratio of solutions A and B). The cell proliferation pattern of F3-effluc cells was compared in the soft 1,800 Pa hydrogel (GPT 1.8 K) and the stiff 5,800 Pa hydrogel (GPT 5.8 K). F3-effluc cells proliferated inside GPT 1.8 K over time, and cells started to migrate out from hydrogel mixture on day 4. Neurosphere-like stem cell clusters were also found on day 4. Scale bar = 10 μm. The arrow represents boundary between the bottom of the well plate and cell/hydrogel mixture. **(b)** In contrast, F3-effluc cells in the stiff GPT 5.8 K proliferated up to 8 day with lower degree than in the soft GPT 1.8 K. Scale bar = 10 μm.

We used the F3-effluc cells that expressed stably the enhanced firefly luciferase (effluc) gene. F3-effluc cells showed more than 85% purity, measured by MACS using anti-CD90.1 microbeads [26]. After the same number of F3-effluc cell was mixed with the soft GPT 1.8 K or the stiff GPT 5.8 K, initial (day 0) phase contrast images showed a distinct boundary of hardening hydrogel matrix containing an evenly distributed F3-effluc cells. F3-effluc cells incorporated within the three-dimensional (3D) GPT 1.8 K were found to have a round shape on day 0 and continuously proliferated until day 2 (Figure 2a). After day 4, the F3-effluc cells proliferated vigorously in 24-well plates, showing a neurosphere-like feature. The F3-effluc cells in the stiff GPT 5.8 K group showed a relatively lower cell growth rate than those in the soft GPT 1.8 K group (Figure 2b). F3-effluc cells proliferated continuously until day 8 in the stiff GPT 5.8 K as well as in the soft GPT 1.8 K.

### *In vitro* luciferase-based evaluation of cell growth rate in the soft and the stiff hydrogels

**Figure 3:**
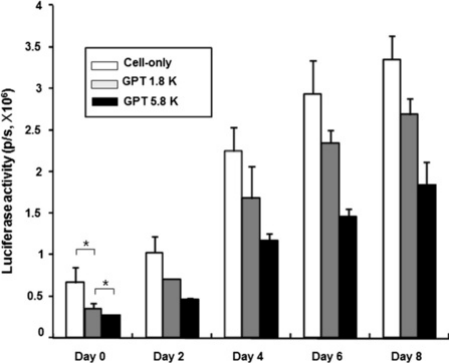
***In vitro*** **luciferase activity versus time in F3-effluc cells incorporated within the soft and stiff hydrogels.** The same number of F3-effluc cells within GPT 1.8 K and GPT 5.8 K was seeded into the six-well plates. The two hydrogel groups and the cell-only group were compared, and in all three groups, cell density increased continuously up to day 8. *P value <0.1.

We compared the proliferation rates of F3-effluc cells within the soft GPT 1.8 K and the stiff GPT 5.8 K over time by examining the luciferase activities of each cell/hydrogel group. All the groups (cell-only, cell/GPT 1.8 K complex, and cell/GPT 5.8 K complex) showed gradually increasing proliferation rate until day 8 (Figure 3). However, the luciferase signals for the cell/hydrogel groups were lower than those for the cell-only group at all time points. To investigate whether the hydrogel matrix influences penetration of d-luciferin, the *in vitro* kinetics of luciferase activity after d-luciferin administration was examined in the soft GPT 1.8 K and the stiff GPT 5.8 K. The F3-effluc cells reached a plateau 15 min after d-luciferin addition in the cell-only group, and the F3-effluc cells in the soft hydrogel time-to-peak extended to 25 min and the cells in the stiff GPT 5.8 K extended to 30 min (Additional file 1: Figure S1). d-Luciferin penetration took more time to reach the cells *in vitro*.

### *In vivo* bioluminescence kinetics of F3-effluc cells within the soft and the stiff hydrogels in the mouse xenograph model

**Figure 4:**
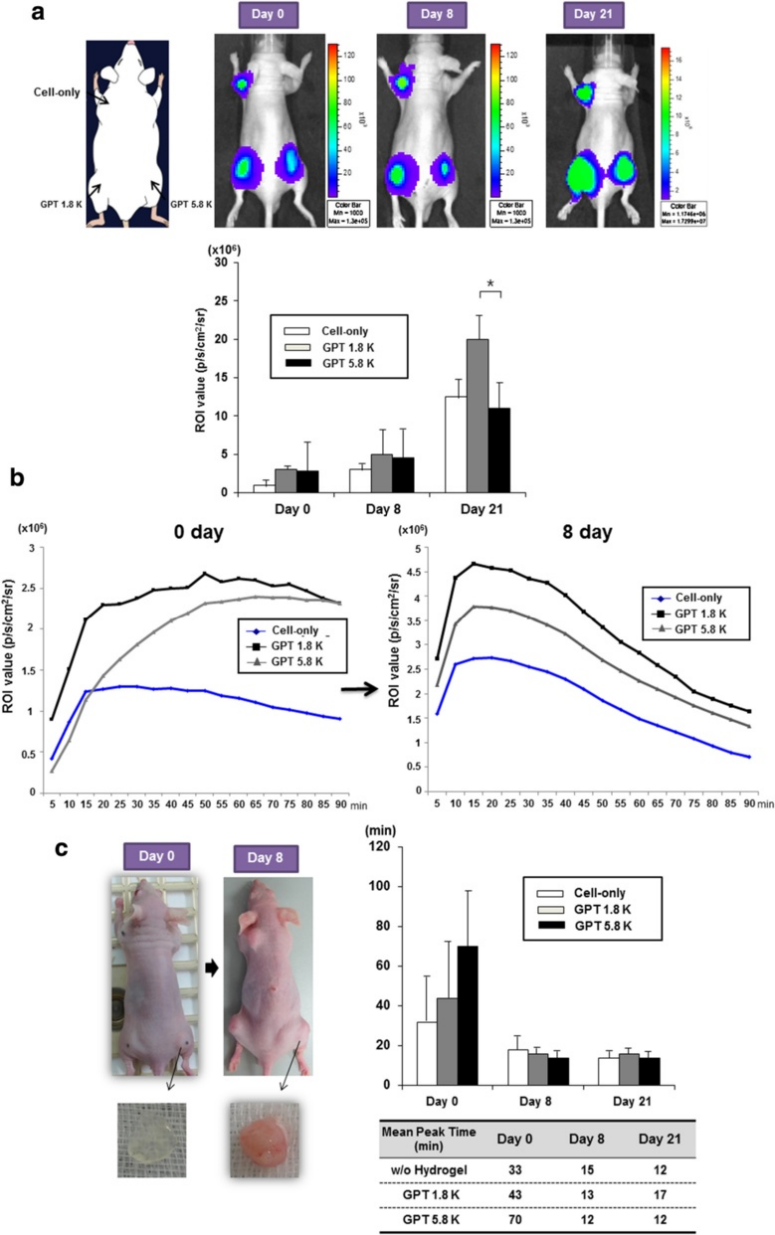
***In vivo*** **bioluminescence imaging of implanted F3-effluc within hydrogel possessing different elasticities in nude mice. (a)** A dual injection syringe containing 1 × 10^6^ F3-effluc cells with HRP and H_2_O_2_ solution was used to produce a rapid gel reaction in *in vivo* environment. The bioluminescence images were acquired at different time points (0, 8, and 21 days) using an IVIS-100 imaging device (*n* = 3). Cell growth in the soft GPT 1.8 K was greater compared with both the cell-only and the stiff GPT 5.8 K groups. *P value <0.1 **(b)** *In vivo* kinetics of luciferase activity was conducted after d-luciferin administration in cell/hydrogel-injected nude mice. Bioluminescence signals were serially measured for 90 min at intervals of 5 min in the same mice. **(c)** The transparent hydrogel containing F3-effluc cells was resected from the thigh of mouse on day 0. The cell/hydrogel complex became pinkish with a large cell mass that proliferated within the complex on day 8. The initial time-to-peak on day 0 was different between the cell-only group and the soft and the stiff hydrogel groups. However, on day 8 and after, times-to-peak became similar for the three groups.

The *in vivo* characteristics of F3-effluc cells incorporated within the soft and the stiff hydrogels were examined on the implanted xenograft in nude mice using a dual injection syringe. A dual hydrogel injection system is composed of two separate syringes. One syringe contains HRP dissolved in GPT solution, and the other syringe contains H_2_O_2_ in GPT solution. F3-effluc cells were loaded in the HRP-containing GPT solution, and then the cells prepared in the dual hydrogel injector were implanted into the thigh of a nude mouse (Additional file 2: Figure S2). The luciferase signals increased in the cell-only and the F3-effluc/hydrogel complex implanted groups until 21 days, showing higher proliferation of F3-effluc cells within the soft GPT 1.8 K than within the stiff GPT 5.8 K or in the cell-only condition (Figure 4a). Cell growth rate was about 1.8-fold higher in the F3-effluc/GPT 1.8 K group than in the F3-effluc/GPT 5.8 K group.

When we examined *in vivo* kinetics of luciferase activity after d-luciferin administration, on day 0, the F3-effluc cells in the cell-only condition showed the fastest time-to-peak for luciferase signal by reaching a plateau at about 30 min. However, the time-to-peak in F3-effluc in the soft GPT 1.8 K group was extended to 50 min, and the time-to-peak in F3-effluc in the stiff GPT 5.8 K group was slower (at 70 min) (Figure 4b).

Interestingly, the activity became very similar among the three groups on day 8, showing times-to-peak of approximately 15 min for all groups (Figure 4b). On day 0, extraction of the cell/hydrogel complex after several hours of operation revealed the transparent cell/hydrogel complex. It became opaque and pinkish due to proliferated F3-effluc cells on day 8 (Figure 4c, left panel). Time-to-peak was shortest in the cell-only group, and that of the soft GPT 1.8 K group was shorter than that of the stiff GPT 5.8 K group on day 0. But on days 8 and 21, they did not show any difference (Figure 4c, right panel).

### Immunohistochemistry of the extracted F3-effluc/hydrogel complex

**Figure 5:**
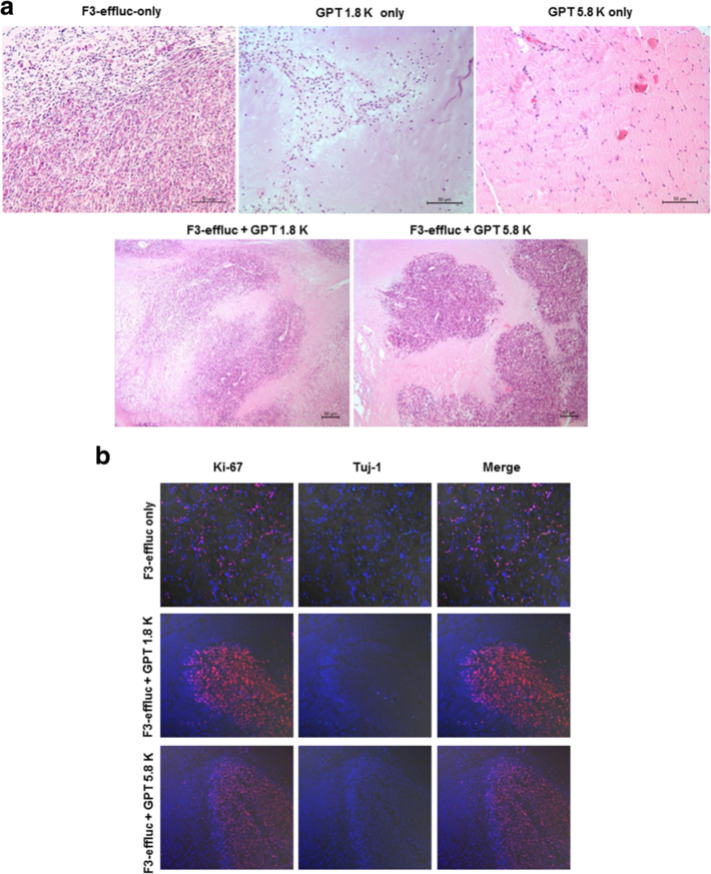
**Immunostaining results from F3-effluc cells encapsulated within the hydrogel matrix. (a)** Hematoxylin and eosin staining on day 21 showed a homogenous distribution in the cell-only group (left upper panel). A small quantity of the host cells was infiltrated into each hydrogel (upper middle and right panel). A partially colonized cell mass (lower two panels) in the hydrogel was found in the soft GPT 1.8 K and the stiff GPT 5.8 K groups. **(b)** Each section acquired from the resected cell mass was stained using different antibodies. The proliferated F3-effluc cells from all groups did not show any Tuj1 (early neuronal marker) expression. In contrast, implanted cells within the hydrogels were highly stained (red color) with the proliferative marker, Ki-67, compared to the cell-only group. The nucleus was counterstained with DAPI (blue color).

On day 21, after the bioluminescence imaging, the cell/hydrogel complex was surgically resected from both thighs of the nude mice. Hematoxylin and eosin (H&E) staining cells were compactly distributed in the cell-only group (Figure 5a). In contrast, colonized cell population was observed in the F3-effluc in the soft GPT 1.8 K and F3-effluc in the stiff GPT 5.8 K sections. In the immunostaining of each section using Tuj-1 (early neuronal marker) and Ki-67 (proliferative marker) specific antibody, Ki-67 was weak in the cell-only sections and was high in F3-effluc cells within the soft GPT 1.8 K and the stiff GPT 5.8 K hydrogels. No Tuj-1-positive cells were observed in sections of any group (Figure 5b).

## Discussion

High-water-content hydrogels with favorable biocompatibility have presented many benefits for various biomedical applications such as drug delivery and stem cell therapy [27–34]. Being highly permeable to oxygen, nutrients, and metabolites, hydrogels can be used as artificial niches for paracrine signaling between cells and the extracellular matrix. In particular, three-dimensional hydrogels play an important role as scaffolds, supporting grafted stem cells and minimizing local inflammation and immunogenic reactions in tissue engineering and regenerative medicine [35–37]. In terms of therapeutics, the use of hydrogels may help grafted stem cells make long-term retention in the injured area by preventing grafted stem cells from uncontrollable migration to the stem cell niche microenvironment.

In this study, gelatin-based biocompatible hydrogels were used whose elasticity can be controlled by varying the concentrations of the cross-linking reagent, and the ability of grafted stem cell proliferation within different types of hydrogels was evaluated *in vivo* as well as *in vitro*. We focused mainly on the *in vivo* behavior of neural stem cells encapsulated with the soft GPT 1.8 K condition. For the future goal of neural stem cell-based therapy in the brain, elasticity of less than 1 kPa, which is similar to that of the intact brain tissue, would have been ideal. However, hydrogels of less than 1 kPa degraded rapidly and was found to be unsuitable for cell culture due to low cross-linking density in culture conditions. Therefore, we chose the GPT 1.8 K that sustains without degradation in culture conditions and possess slightly harder properties than the brain tissue (0.1 to 1 kPa) and then compared its performance to the stiff GPT 5.8 K [17–19].

In our results, the bright-field microscopy data showed that cell growth rate of F3-effluc cells incorporated within the soft GPT 1.8 K was higher than that of the cells within the stiff GPT 5.8 K. Adherent F3-effluc cells in 3D culture condition began to migrate out from the hydrogel boundary around 4 days after mixing the cells with the hydrogel of both types. We also observed neurosphere-like features for F3-effluc cells inside the soft GPT 1.8 K with dense, compact cell clusters at 4 days. These were not observed in the GPT 5.8 K at 4 days, which demonstrated that the soft hydrogel environment might have been more suitable for *in vitro* cell proliferation (Figure 2a,b). We found that luciferase signals were lower in the hydrogel groups than in the cell-only group and the luciferase activity in the cells in the stiff GPT 5.8 K was lower than that in the soft GPT 1.8 K in the same conditions. We speculate that d-luciferin is less able to penetrate the compact and stiff hydrogel according due to the elastic properties *in vitro*. Considering that the *in vivo* condition is more adverse for the d-luciferin to penetrate the cell/hydrogel complex, we investigated the *in vivo* kinetics luciferase activity after d-luciferin administration and found that the cell-only group showed more rapidly increasing luciferase activity on day 0, compared with both hydrogel groups. These results support our idea that the presence of hydrogel makes d-luciferin reach the cells inside more slowly. We suspect that the stiff GPT 5.8 K is less permeable to the d-luciferin substrate. On day 0, time-to-peak was the longest in the stiff GPT 5.8 K *in vivo*. However, on day 8 and day 21, degradation of the implanted hydrogel associated with proliferating F3-effluc cells induced similar kinetics of luciferase activity in both types of hydrogel scaffolds in that d-luciferin penetration was not different between the soft and the stiff hydrogels even though experimental scale error bar was high on day 0, possibly owing to initial injection techniques or injection depth (Figure 4b,c).

Although the aforementioned *in vivo*d-luciferin kinetic study showed a similar time-to-peak value between the soft and stiff hydrogels on day 21, we observed the higher proliferation of F3-effluc cells within the soft GPT 1.8 K, demonstrating that the soft microenvironment (1.8 K Pa) that is similar to the mechanical strength of the intact brain is more suitable for neural stem cell growth compared to the stiff environment (Figure 4a). Since the soft GPT 1.8 K has higher water content and low cross-linking density, *in vivo* degradation would have been more rapid than in the stiff GPT 5.8 K and factors such as nutrients and hormones would have better chance to reach the cells.

Our *in vivo* molecular imaging technique enabled us to monitor survival and proliferation of grafted cells paired with biomaterials such as poly-l-lactic acid (PLLA) in a non-invasive manner in living subjects [38]. This study was also the case with cell/hydrogel complexes implanted to the nude mice. Image-based studies in the tissue engineering field easily provide essential information about how many scaffold-encapsulated cells were implanted at the beginning and how long the implanted cells could sustain within the scaffold *in vivo*. In particular, for cell-based therapies, such methodologies can be used as a means to determine the optimal cell number to be implanted.

The *in vivo* optical imaging to examine the cell/hydrogel implantation has been reported by several groups, who assessed the effectiveness of bioluminescence techniques by monitoring viable stem cells within the hydrogel *in vivo* [39,40]. In this study, studies for evaluating critical parameters such as the mechanical strength of hydrogel were easily conducted *in vivo* by molecular optical imaging techniques. We could determine which hydrogel was more appropriate for more cells to survive and proliferate in the cell/hydrogel implants.

Surface-functionalized hydrogels, such as the RGD-cross-linked hydrogel, were introduced to enhance cell proliferation [40–42]. The grafted stem cells distributed inside MMP-sensitive peptide-modified hydrogels exhibited enhanced expression of early cardiac cell-related transcription factor and induced cardioprogenitor differentiation [41]. RGD-modified hydrogel mimicking the *in vivo* extracellular matrix structure was applied to an animal model, showing functional improvement in spinal cord injury [42]. Taking these examples into consideration, hydrogel could be one of the most attractive biomaterials in terms of biocompatibility and biodegradability. However, so as to maximize the cell survival *in vivo*, we need to choose the appropriate hydrogel among the wide variety available. Thus, the determination of the optimal elasticity for a hydrogel is of great importance, and bioluminescence *in vivo* imaging was the study of choice in this investigation. The *in vivo* characteristics of biomimetic hydrogels of different elasticities could easily be evaluated by *in vivo* molecular imaging. Using this invaluable information from *in vivo* molecular imaging, hydrogel-based cell implantation therapy will eventually enhance the efficacy of regenerative therapy.

## Conclusions

This work establishes the efficacy of *in vivo* monitoring of the viability of implanted neural stem cells, incorporated within different types of bioactive hydrogel. It was found that the neural stem cells within GPT 1.8 K (soft gel type) showed a higher proliferation rate than those in GPT 5.8 K (stiff gel type), in subcutaneously injected mice. From kinetic studies using a luciferase-based optical imaging technique, different bioluminescence signal dynamics were found for the GPT 1.8 K and GPT 5.8 K, both *in vitro* and *in vivo*. Our results provide useful information to determine the desired hydrogel elasticity or the optimal optical imaging conditions for hydrogel-encapsulated implanted cells. The developed methodology, based on *in vivo* imaging, will help to enhance the therapeutic efficacy of stem cell therapy by providing essential information for the treatment of neurological disease.

## Additional files

Additional file 1: Figure S1. In vitro kinetic analysis of luciferase activity after d-luciferin administration. The luciferase activity was measured for 95 min at intervals of 5 min in F3-effluc cell-only, the soft GPT 1.8 K/cell, and the stiff GPT 5.8 K/cell groups. The overall luciferase intensity curve over time was a little different for the three cell groups. Additional file 2: Figure S2. A dual hydrogel injection system was adopted to implant the cell/hydrogel complex. Two separate syringes in the dual hydrogel injector contain HRP dissolved in GPT solution and H_2_O_2_ in GPT solution, respectively. Cells were loaded in the GPT solution containing HRP. A dual hydrogel injector containing cells was implanted into the thigh of a mouse.
