# Supplementary figures and images for: In vivo bioluminescence imaging for viable human neural stem cells incorporated within in situ gelatin hydrogels
Source: EJNMMI Res. 2014 Nov 12;4:61. doi: 10.1186/s13550-014-0061-3 (PMC4452629; doi:10.1186/s13550-014-0061-3)

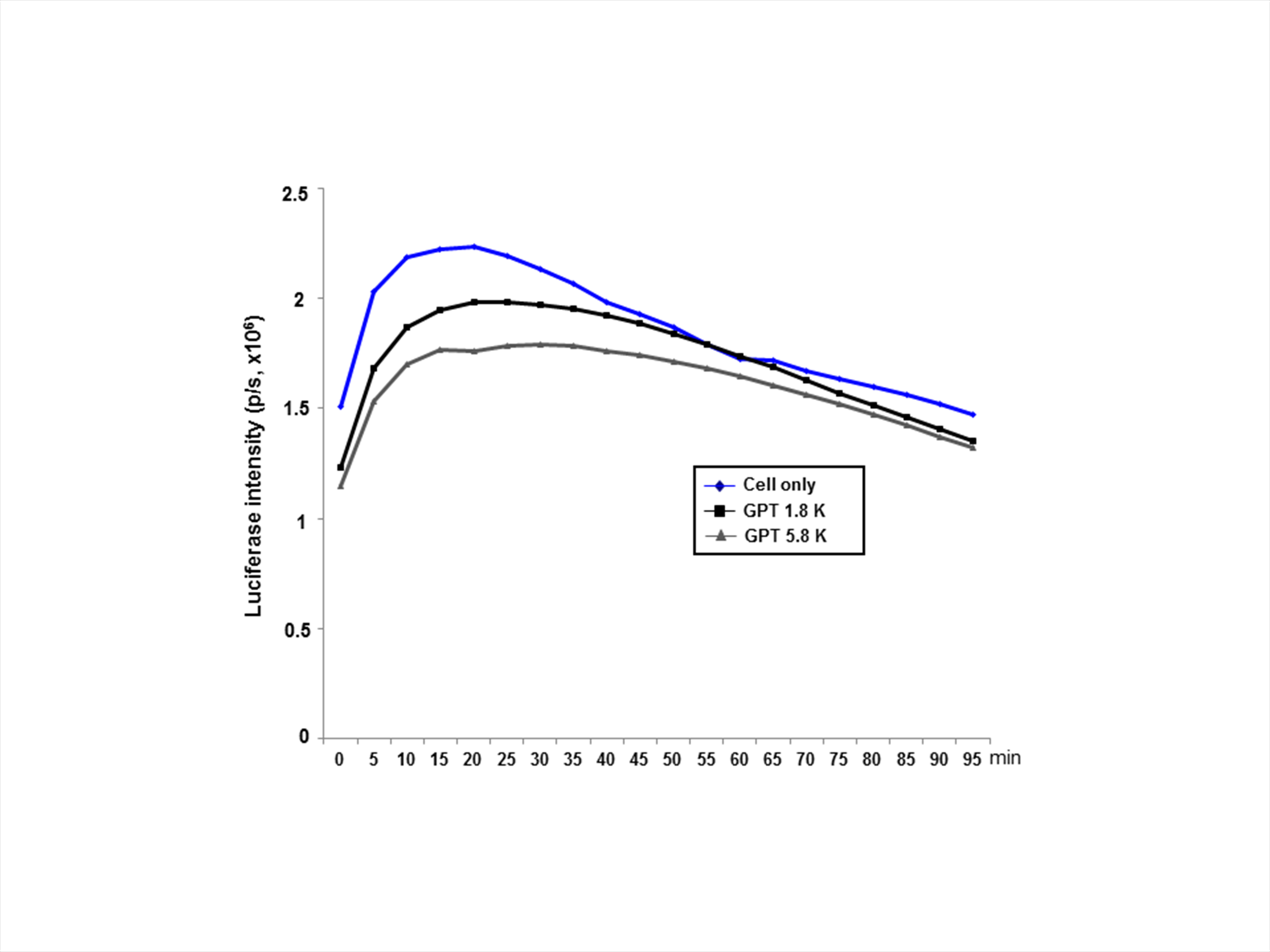

Supplement: Additional file 1: Figure S1. — In vitro kinetic analysis of luciferase activity after d-luciferin administration. The luciferase activity was measured for 95 min at intervals of 5 min in F3-effluc cell-only, the soft GPT 1.8 K/cell, and the stiff GPT 5.8 K/cell groups. The overall luciferase intensity curve over time was a little different for the three cell groups. [file 13550_2014_61_MOESM1_ESM.tiff]

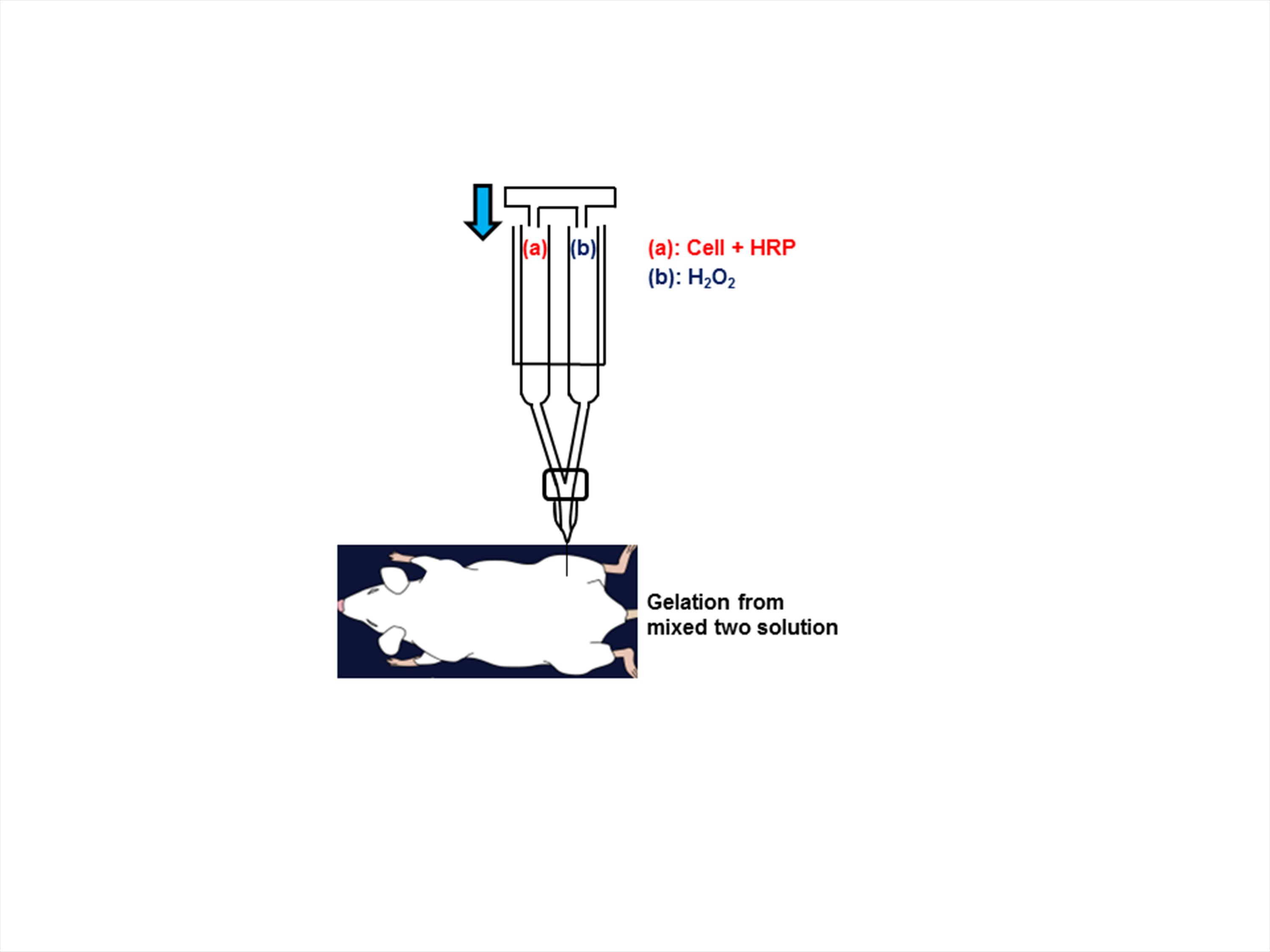

Supplement: Additional file 2: Figure S2. — A dual hydrogel injection system was adopted to implant the cell/hydrogel complex. Two separate syringes in the dual hydrogel injector contain HRP dissolved in GPT solution and H2O2 in GPT solution, respectively. Cells were loaded in the GPT solution containing HRP. A dual hydrogel injector containing cells was implanted into the thigh of a mouse. [file 13550_2014_61_MOESM2_ESM.tiff]
